# Cellular Recovery and Therapeutic Rechallenge After Cancer Therapy-Induced Kidney Injury: Mechanistic Insights and Clinical Implications

**DOI:** 10.3390/cells15171563

**Published:** 2026-08-28

**Authors:** Yosuke Dotsu, Kazumasa Akagi, Noritaka Honda, Midori Matsuo, Hirokazu Taniguchi, Shinnosuke Takemoto, Tomoya Nishino, Hiroshi Mukae

**Affiliations:** 1 Department of Respiratory Medicine, Nagasaki University Hospital, Nagasaki 852-8501, Japan; kaakagi@nagasaki-u.ac.jp (K.A.); nhonda@nagasaki-u.ac.jp (N.H.); mi-shimada@nagasaki-u.ac.jp (M.M.); hirokazu_pc@nagasaki-u.ac.jp (H.T.); shinnosuke-takemoto@nagasaki-u.ac.jp (S.T.); 2 Clinical Oncology Center, Nagasaki University Hospital, Nagasaki 852-8501, Japan; 3 Clinical Research Center, Nagasaki University Hospital, Nagasaki 852-8501, Japan; 4 Clinical Genomics Center, Nagasaki University Hospital, Nagasaki 852-8501, Japan; 5 Department of Nephrology, Nagasaki University Hospital, Nagasaki 852-8501, Japan; tnishino@nagasaki-u.ac.jp; 6 Nagasaki Harbor Medical Center, Nagasaki 850-8555, Japan; hmukae@nagasaki-u.ac.jp

**Keywords:** onco-nephrology, therapeutic rechallenge, acute kidney injury, biological kidney recovery

## Abstract

**Highlights:**

**What are the main findings?**

- Biological kidney recovery extends beyond conventional recovery of kidney function.
- Adaptive and maladaptive repair determine renal resilience after therapy-related AKI.

**What are the implications of the main findings?**

- Therapeutic rechallenge should integrate biological recovery with oncologic benefit–risk assessment.
- Emerging biomarkers may enable biologically informed precision onco-nephrology.

**Abstract:**

Cancer therapy-related acute kidney injury has become an increasingly common challenge as modern treatments prolong survival and increase exposure to potentially nephrotoxic therapies. Decisions regarding therapeutic rechallenge have relied on normalizing serum creatinine and recovering estimated glomerular filtration rate, despite growing evidence that biochemical recovery does not necessarily indicate restoration of kidney integrity or resilience. In this review, we propose biological kidney recovery as a conceptual framework that integrates mechanisms of kidney injury and repair (adaptive and maladaptive) with emerging biomarkers and therapeutic rechallenge. We first summarize the distinct mechanisms of kidney injury induced by platinum-based chemotherapy, immune checkpoint inhibitors, and vascular endothelial growth factor pathway inhibitors, highlighting how these differences influence subsequent repair. We then discuss the cellular and metabolic processes underlying adaptive repair, the transition to maladaptive remodeling, and current approaches for assessing biological recovery through pathology, biomarkers, and multi-omics technologies. Finally, we present a practical framework for individualized therapeutic rechallenge based on an integrated assessment of kidney-, tumor-, and patient-related factors and outline future directions for precision onco-nephrology. By shifting the focus from filtration alone to biological recovery, this framework enables more informed therapeutic rechallenge aimed at preserving both oncologic efficacy and long-term kidney health.

## 1. Introduction

The therapeutic landscape in oncology has changed substantially over the past decade. In addition to conventional cytotoxic chemotherapy, immune checkpoint inhibitors, molecularly targeted agents, antibody–drug conjugates, and increasingly complex combination regimens have expanded treatment options and prolonged survival for patients with various malignancies. An inevitable consequence has been an increased recognition of treatment-related kidney injury [1,2]. Kidney toxicity associated with cancer therapy encompasses a heterogeneous spectrum of injuries, including acute tubular injury, acute interstitial nephritis, thrombotic microangiopathy, glomerular disease, and other forms of vascular and endothelial damage [1]. Platinum compounds remain among the best-established causes of direct tubular toxicity [3], whereas immune checkpoint inhibitors and vascular endothelial growth factor (VEGF) pathway inhibitors produce distinct patterns of injury driven predominantly by immune dysregulation and disruption of endothelial homeostasis, respectively [4,5]. These mechanisms may coexist in patients exposed to sequential treatments, combination regimens, concomitant nephrotoxic medications, volume depletion, or systemic complications. Therefore, identifying the dominant mechanism is crucial not only for diagnosis and initial management but also for predicting the consequences of subsequent drug exposure.

Acute kidney injury (AKI) during cancer treatment poses a major dilemma for clinicians. Permanent discontinuation of the suspected agent may compromise tumor control, particularly when the treatment is highly effective or when alternatives are limited. Conversely, resuming therapy may lead to recurrent injury, irreversible nephron loss, or progression to chronic kidney disease [1,2]. In routine practice, decisions regarding treatment resumption are typically based on improvements in serum creatinine or estimated glomerular filtration rate (eGFR). However, recovery of filtration does not necessarily indicate that cellular and tissue processes initiated by AKI have resolved.

Following injury, surviving tubular epithelial cells undergo coordinated changes in their phenotype, proliferation, metabolism, mitochondrial function, and intercellular signaling [6,7]. These responses may restore epithelial integrity and tissue homeostasis; however, they can also be incomplete, resulting in persistent injury-associated cellular states, inflammation, microvascular dysfunction, or fibrotic remodeling [8]. The recovering kidney may therefore regain sufficient filtration to normalize standard laboratory parameters while remaining vulnerable to further stressors. For the purposes of this review, we define biological kidney recovery as the restoration of cellular integrity, metabolic competence, inflammatory and vascular homeostasis, and tissue resilience sufficient to withstand renewed therapeutic stress, beyond the apparent recovery of glomerular filtration. This working definition is multidimensional and cannot currently be captured by a single clinical measurement. Rather, it may be approximated by integrating conventional kidney function, resolution of the original injury phenotype, relevant pathological findings, and, where available, markers of residual cellular or molecular injury. Importantly, no validated threshold or composite criterion currently exists to establish biological kidney recovery or to determine readiness for therapeutic rechallenge. Thus, biological kidney recovery should be regarded as a conceptual framework rather than an established clinical endpoint, but it may help explain why patients with similar biochemical recovery exhibit different outcomes after rechallenge with nephrotoxic therapy.

Clinical evidence regarding therapeutic rechallenge after cancer therapy-related AKI remains limited. Direct studies characterizing the cellular and molecular processes of kidney repair specifically after cancer therapy-related AKI are also sparse. Accordingly, throughout this review, we distinguish cancer therapy–specific evidence from mechanistic concepts extrapolated from general clinical and experimental AKI. Most available data come from retrospective cohorts, small case series, or case reports, and the biological quality of kidney recovery is rarely incorporated into treatment decisions. Moreover, there is currently no validated biomarker to determine whether the kidney has recovered sufficiently to tolerate an anticancer rechallenge. Although retrospective studies have demonstrated the oncologic feasibility of platinum retreatment in selected cancer populations [9,10], these studies were not designed to evaluate the renal safety of rechallenge specifically after platinum-associated AKI. Our recent report of cisplatin administration after recovery from biopsy-proven carboplatin-associated acute tubular necrosis therefore represents a clinically illustrative, hypothesis-generating observation rather than evidence supporting the general safety of platinum rechallenge [11]. As a single case, it cannot establish patient-selection criteria, optimal timing, or the safety of rechallenge in broader clinical practice. Nevertheless, it highlights a broader question that is increasingly relevant in contemporary oncology: how should clinicians determine whether kidney recovery is sufficient to justify the resumption of an important anticancer treatment?

**Figure 1:**
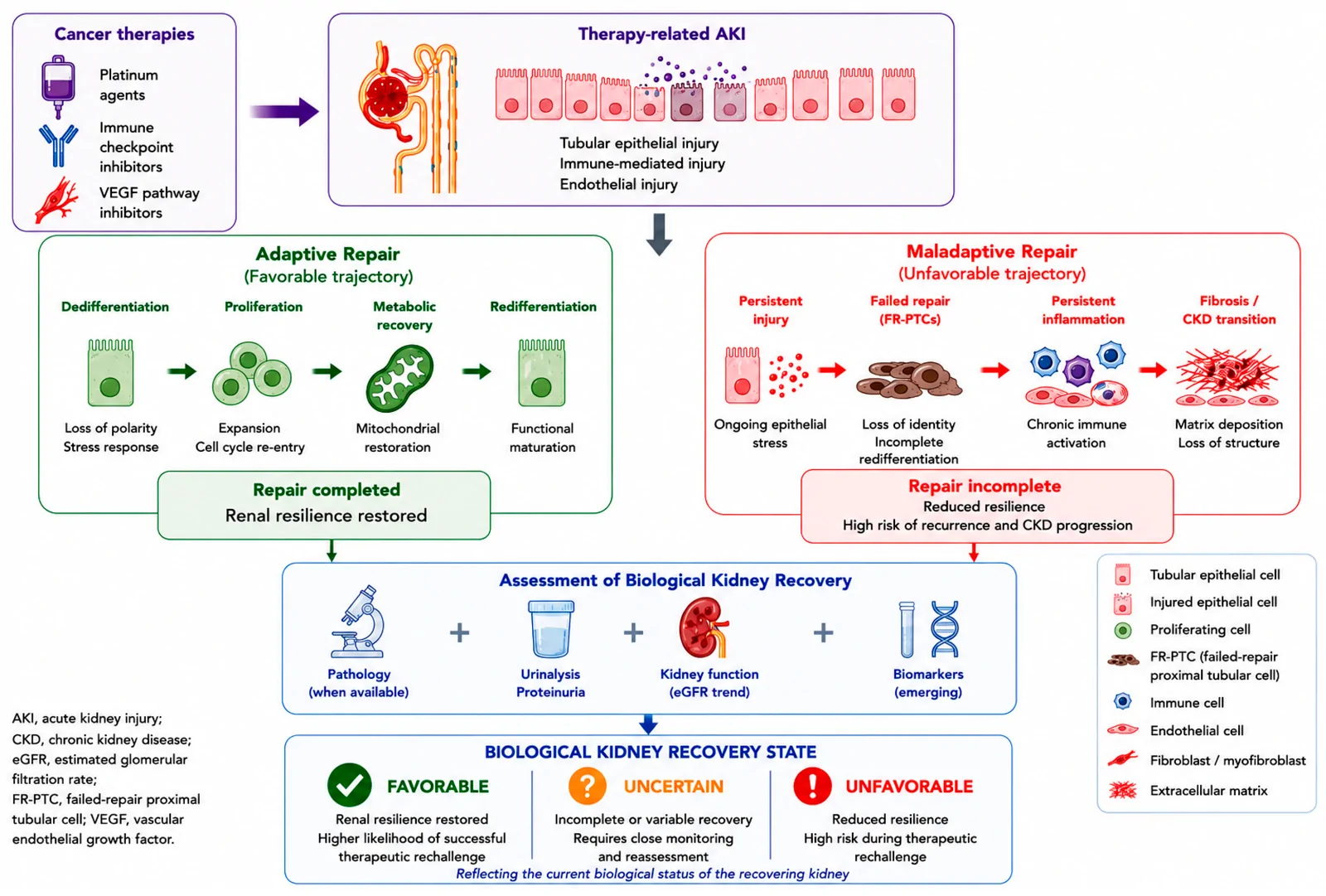
Conceptual framework of biological kidney recovery after cancer therapy-related acute kidney injury. Cancer therapies, including platinum agents, immune checkpoint inhibitors, and VEGF pathway inhibitors, induce distinct forms of therapy-related acute kidney injury (AKI), including tubular epithelial injury, immune-mediated injury, and endothelial injury. Following AKI, kidney repair may proceed along adaptive or maladaptive trajectories. Adaptive repair is characterized by transient epithelial dedifferentiation, proliferation, metabolic recovery, and redifferentiation, leading to restoration of renal resilience. In contrast, maladaptive repair is associated with persistent epithelial injury, failed-repair proximal tubular cells (FR-PTCs), chronic inflammation, fibrosis, and progression toward chronic kidney disease (CKD), resulting in incomplete repair and reduced renal resilience. Biological kidney recovery is assessed by integrating pathology (when available), urinalysis and proteinuria, kidney function, and emerging biomarkers. These complementary findings inform a provisional biological recovery state—favorable, uncertain, or unfavorable—which provides the kidney-specific biological input for subsequent clinical decision-making. Figure partially created in BioRender. Taniguchi, H. (2026) https://BioRender.com/p7dooda (accessed on 25 August 2026). The final illustration was generated and refined using ChatGPT image generation (OpenAI; GPT 5.6-Sol). The scientific concept, content, interpretation, and overall structure were developed and verified by the authors.

Section 2, Section 3, Section 4, Section 5 and Section 6 examine cancer therapy-related kidney injury through the interconnected processes of injury, adaptive and maladaptive repair, biomarker assessment, and therapeutic rechallenge. We then consider how pathological, biological, and clinical information can be integrated to individualize treatment resumption while distinguishing established clinical evidence from mechanistic inference. The conceptual framework is illustrated in Figure 1.

## 2. Cellular Mechanisms of Kidney Injury

Cancer therapy–induced kidney injury encompasses a heterogeneous group of injuries that vary by therapeutic class, the affected kidney compartment, and the dominant mechanism of injury [1,2]. These mechanisms often overlap in patients receiving combination regimens, sequential therapies, or concomitant nephrotoxic medications. However, identifying the predominant pattern remains clinically important because direct tubular toxicity, immune-mediated inflammation, and glomerular endothelial injury differ in their pathological features, reversibility, and response to treatment re-exposure.

### 2.1. Platinum-Associated Kidney Injury

Platinum compounds are among the best-established nephrotoxic anticancer agents [3]. Cisplatin is the leading cause of clinically significant platinum-associated kidney injury, although carboplatin and other platinum agents may also be implicated, particularly in patients with reduced kidney reserve, dehydration, prior kidney injury, or exposure to other nephrotoxins. The proximal tubule is the primary site of damage because platinum accumulates within tubular epithelial cells, which are highly dependent on mitochondrial energy production [3,12].

Intracellular platinum induces DNA damage and activates cellular stress pathways [12,13]. Mitochondrial injury reduces ATP production and increases the production of reactive oxygen species, thereby impairing energy-dependent transport and redox homeostasis [13,14]. Oxidative stress promotes lipid peroxidation, protein damage, and membrane disruption [3,15]. The resulting epithelial injury may lead to loss of the brush border, tubular obstruction, cell death, and localized inflammatory signaling.

Platinum nephrotoxicity is not solely dose-dependent. Hydration status, magnesium depletion, cumulative exposure, baseline kidney reserve, and prior kidney injury all affect susceptibility [16]. Regulated cell death pathways may also contribute. Experimental studies have implicated ferroptosis—an iron-dependent process driven by lipid peroxidation—in platinum-associated tubular injury, highlighting its interaction with mitochondrial dysfunction and oxidative stress [17]. The relative contribution of these pathways likely varies among patients and injury stages.

### 2.2. Immune Checkpoint Inhibitor-Associated Kidney Injury

Immune checkpoint inhibitors cause a distinct pattern of kidney injury driven primarily by immune dysregulation rather than by direct tubular cytotoxicity [5]. Acute interstitial nephritis is the most common histologic lesion, although the full clinicopathologic spectrum is broader [18].

PD-1, PD-L1, or CTLA-4 blockade can disrupt peripheral immune tolerance and activate autoreactive T cells [5,19]. These cells may recognize kidney antigens or drug-associated antigens, resulting in interstitial inflammation and tubulitis. Proton pump inhibitors, antibiotics, and nonsteroidal anti-inflammatory drugs are common concurrent exposures and may serve as additional immune triggers, complicating causal attribution [18].

Kidney biopsies typically reveal interstitial inflammatory infiltrates with varying degrees of tubular involvement. Glomerular lesions and other immune-mediated manifestations have also been reported, although they are less common [4]. Therefore, the biological consequence is not limited to a loss of filtration. A persistent underlying immune response may remain clinically relevant even after serum creatinine levels improve.

### 2.3. VEGF Pathway Inhibition

Inhibition of the VEGF pathway causes nephrotoxicity by disrupting glomerular endothelial and podocyte homeostasis. VEGF signaling is essential for maintaining endothelial fenestrae, capillary integrity, and functional interactions between podocytes and the glomerular filtration barrier [20,21]. Disruption of this pathway may result in hypertension, proteinuria, impaired kidney function, and thrombotic microangiopathy [22,23,24].

The predominant lesion is vascular rather than tubular. Loss of endothelial fenestrae, capillary wall injury, and activation of prothrombotic pathways compromise glomerular microvascular function [21,23]. Clinical manifestations range from asymptomatic proteinuria and exacerbation of hypertension to nephrotic syndrome, kidney-limited thrombotic microangiopathy, and clinically significant AKI [22,24].

Kidney function may improve after dose reduction or treatment discontinuation; however, normalization of serum creatinine or reduction in proteinuria does not necessarily indicate complete microvascular recovery. Therefore, persistent endothelial or podocyte injury may influence the risk of recurrent toxicity after re-exposure [21,22]. Assessment should include blood pressure, proteinuria, and kidney function and, when clinically indicated, laboratory evidence of microangiopathy.

### 2.4. Clinical Significance of the Injury Mechanism

These three patterns illustrate why cancer therapy–induced kidney injury should not be managed with a uniform approach [1,25]. Similar increases in serum creatinine levels may reflect proximal tubular damage during platinum therapy, interstitial inflammation during immune checkpoint inhibitor therapy, or glomerular endothelial injury during VEGF pathway blockade. Each lesion has distinct biological requirements for recovery, including restoration of epithelial and mitochondrial competence following tubular injury, resolution of immune activation after interstitial nephritis, and recovery of microvascular and filtration barrier integrity following endothelial damage.

This distinction also influences the interpretation of apparent kidney recovery. Filtration may improve before the affected cellular compartment achieves homeostasis. Therefore, the safety of therapeutic rechallenge depends not only on the extent of creatinine normalization but also on whether the original pathological process has resolved. Clinical evidence for rechallenge is most robust for ICI-associated AKI, with retrospective data and expert recommendations supporting individualized retreatment after kidney recovery in selected patients [26,27]. When the cause of AKI remains uncertain and biopsy results are expected to alter clinical management, kidney biopsy can differentiate between tubular injury, interstitial nephritis, glomerular disease, and endothelial lesions [28]. Therefore, characterizing the initial injury is the first step in understanding the subsequent course of kidney repair. Section 3 examines how injured renal tissue restores its epithelial structure and function and why apparent recovery may remain biologically incomplete. The major patterns of cancer therapy-related kidney injury and their implications for kidney recovery and therapeutic rechallenge are summarized in Table 1.

## 3. Cellular Mechanisms of Kidney Repair

Kidney recovery after AKI requires coordinated restoration of epithelial architecture, cellular metabolism, and the surrounding tissue environment [29,30]. Importantly, much of the mechanistic understanding of adaptive and maladaptive kidney repair discussed below derives from experimental models of ischemic, toxic, or other forms of AKI and from studies of human kidney disease rather than specifically from cancer therapy-related AKI. Direct evidence demonstrating these repair trajectories after anticancer therapy–induced kidney injury remains limited. Accordingly, the mechanisms discussed below should be interpreted as a biologically plausible framework extrapolated from the broader AKI literature unless cancer therapy–specific evidence is explicitly indicated. The extent to which individual anticancer agents reproduce these cellular trajectories in patients remains an important area for future investigation. The proximal tubule has considerable regenerative capacity, but the outcome depends on the severity and duration of injury, the number and condition of surviving epithelial cells, the preservation of the tubular basement membrane, and signals from the inflammatory, vascular, and stromal compartments [8,31]. Therefore, recovery is determined not only by the number of surviving cells but also by their ability to complete the transition back to a mature, functionally competent state.

### 3.1. Cellular Origin of Tubular Repair

Lineage-tracing studies indicate that the repair of injured proximal tubules is predominantly mediated by epithelial cells that survive the initial insult [32]. These cells undergo phenotypic adaptation, re-enter the cell cycle, and repopulate the denuded areas of the tubular lining. Although some epithelial subpopulations may display greater proliferative capacity, current evidence does not support a single dedicated extratubular stem cell population as the primary source of repair [29,32,33].

The extent of viable epithelium and the integrity of the tubular basement membrane strongly influence this response. When both are preserved, local migration, proliferation, and redifferentiation can restore tubular continuity. Extensive epithelial loss or disruption of the basement membrane limits this process and increases the likelihood of incomplete repair [8,31].

### 3.2. Transient Dedifferentiation and Cell Cycle Re-Entry

Surviving tubular epithelial cells enter a transient repair state following injury. They downregulate mature transport programs, lose apicobasal polarity, remodel the cytoskeleton, and activate developmental and stress-response transcriptional pathways [7,29,34]. This response is often termed dedifferentiation, although it is better understood as a reversible change in cellular state rather than as a loss of epithelial identity.

The reparative phenotype allows cells to migrate across the denuded basement membrane and re-enter the cell cycle. Pathways involving epidermal growth factor, hepatocyte growth factor, Wnt/β-catenin, Notch, Hippo-YAP, and SOX9 contribute to epithelial survival, plasticity, and proliferation [29,33]. Their effects depend on duration and context. Transient activation supports repair, whereas sustained signaling can delay redifferentiation and promote inflammatory or profibrotic responses.

However, cell cycle entry alone is insufficient. Successful repair also requires timely withdrawal from the cell cycle and re-establishment of epithelial polarity, junctional organization, and segment-specific transport functions [29]. Cells that fail to complete this sequence may persist in an injury-associated intermediate state [7,34].

### 3.3. Restoration of Tubular Architecture

Proliferation replaces the epithelial cells lost during the acute phase, but the magnitude of the proliferative response does not determine the quality of repair [32,33]. Cell division must be coordinated with migration, extracellular matrix remodeling, and the restoration of cell–cell and cell–matrix interactions.

Reconstructing the epithelial barrier requires reestablishing tight and adherens junctions, recovering apicobasal polarity, restoring the brush border, and re-expressing transporters specific to the affected nephron segment [29]. These structural events may proceed at a different rate from the recovery of filtration. Therefore, the morphological closure of an epithelial defect is an intermediate step rather than proof that tubular function has been restored.

### 3.4. Metabolic and Mitochondrial Recovery

Proximal tubular cells rely heavily on mitochondrial oxidative metabolism to sustain active solute transport. Mitochondrial injury during AKI compromises both cell survival and the ability of surviving cells to regain mature tubular function [35,36]. Therefore, the reparative phase requires the recovery of cellular energy production and the restoration of epithelial structure.

Restoring mitochondrial homeostasis involves mitochondrial biogenesis, mitophagy, recovering membrane potential and respiratory capacity, and remodeling the mitochondrial network [35,36,37]. These processes remove damaged organelles and rebuild a functional mitochondrial pool. Experimental activation of the SIRT1–PGC-1α pathway accelerates mitochondrial and tubular recovery after ischemia–reperfusion injury, supporting a direct role for mitochondrial biogenesis in kidney repair [38].

This balance can be easily disrupted. Inadequate removal of dysfunctional mitochondria perpetuates oxidative stress, whereas excessive loss of mitochondria limits ATP production. Persistent suppression of fatty acid oxidation and oxidative phosphorylation is characteristic of an incompletely repaired tubular epithelium and has been linked to the transition from AKI to chronic kidney disease [36,37]. Therefore, metabolic recovery is part of redifferentiation, not merely a secondary consequence of structural repair.

### 3.5. Redifferentiation and Functional Recovery

After epithelial expansion, repairing cells must reacquire the molecular and structural features of the mature tubular epithelium. Redifferentiation requires reacquisition of segment-specific gene expression and transport function [29]. Without this step, an anatomically intact epithelium may remain functionally impaired.

Single-cell and single-nucleus transcriptomic studies, predominantly performed in experimental AKI models, have shown that this transition is heterogeneous [7,34]. Many cells return to a differentiated state, whereas others retain injury-associated transcriptional programs. Residual abnormalities may include impaired transport, altered metabolism, persistent stress signaling, and a reduced capacity to respond to subsequent insults. These differences are not readily captured by conventional filtration markers and become especially relevant when the kidney is re-exposed to nephrotoxic agents.

### 3.6. Cellular Senescence and Persistent Cell Cycle Arrest

Cell cycle arrest is a normal component of the early response to injury. Transient arrest can prevent the replication of damaged DNA and allow the repair or removal of irreversibly injured cells [39,40]. Some senescence-associated signals may also contribute to tissue remodeling and the recruitment of immune cells during the acute phase.

Persistent senescence has several consequences. Senescent epithelial cells develop a senescence-associated secretory phenotype and release inflammatory and profibrotic mediators that affect neighboring epithelial, immune, endothelial, and stromal cells [39]. Similarly, persistent G2/M arrest has been associated with profibrotic signaling after AKI [41]. These states overlap but should not be considered interchangeable; senescence, G2/M arrest, and failed-repair epithelial phenotypes are characterized by different biological features and experimental criteria.

Their interpretation also depends on timing. Markers associated with senescence or cell cycle arrest may appear transiently during successful repair, while sustained expression is more consistent with a failure to complete the repair program [39]. No single marker is sufficient to distinguish between these states.

### 3.7. From Adaptive to Maladaptive Repair

Adaptive repair ends when surviving epithelial cells recover their mature structure, metabolism, and function. A subset of injured cells fails to complete this process. Single-cell studies have identified persistent epithelial populations that retain injury-associated transcriptional programs after the acute event has subsided [7,34]. These observations arise largely from experimental and non-cancer AKI settings and provide a mechanistic model, rather than direct evidence, for incomplete recovery after cancer therapy-related AKI. These findings support a model in which repair progresses through a continuum of cellular states, rather than as a simple transition from injury to recovery. Persistent epithelial stress, incomplete redifferentiation, metabolic dysfunction, and abnormal intercellular signaling are the initiating factors for maladaptive remodeling. Section 4 examines how these unresolved epithelial states sustain inflammation, activate stromal responses, and contribute to fibrosis and the AKI-to-CKD transition.

## 4. Maladaptive Repair

### 4.1. Persistence of Failed-Repair Epithelial States

Single-cell and single-nucleus transcriptomic analyses have revealed heterogeneous epithelial trajectories after AKI, including persistent injury-associated states that fail to complete the normal repair program [7,34,42]. Among these populations, failed-repair proximal tubular cells (FR-PTCs) have emerged as a defining feature of maladaptive repair. First identified by Kirita et al. in experimental AKI, FR-PTCs are characterized by the persistent activation of injury-response programs, incomplete recovery of differentiated tubular functions, and sustained expression of inflammatory and profibrotic mediators despite resolution of the acute insult [7]. Similar epithelial states have been identified across multiple experimental models and human kidney diseases, indicating that they represent a conserved cellular response rather than a model-specific phenomenon [34,42]. However, FR-PTCs have not yet been established as a defined cellular population or prognostic marker in cancer therapy-related AKI. Their relevance to therapeutic rechallenge therefore remains mechanistically compelling but currently inferential.

Translating the FR-PTC concept into routine pathology remains challenging because these cells are defined primarily by their transcriptional state rather than by a validated histological marker. Tubular VCAM-1 has emerged as a candidate tissue surrogate, as VCAM-1-positive proximal tubular cells localize to persistently injured tubular populations in experimental AKI [7]. KIM-1 can provide complementary evidence of tubular injury but is not specific for failed repair, because its expression is prominent during acute injury and may be absent from some persistent FR-PTCs. A provisional pathological phenotype might therefore combine tubular VCAM-1 positivity with evidence of epithelial injury/dedifferentiation and loss of mature tubular features. However, no immunohistochemical panel has been validated to identify FR-PTCs or predict kidney recovery or rechallenge outcomes, particularly after cancer therapy-related AKI.

FR-PTCs are defined by cellular state rather than by lineage. They should not be regarded simply as tubular cells with delayed regeneration, but as a stable epithelial phenotype that actively shapes the surrounding kidney microenvironment. Through their sustained production of cytokines, chemokines, growth factors, and other signaling molecules, these cells continuously communicate with endothelial cells, fibroblasts, and infiltrating immune cells, thereby influencing the trajectory of tissue repair [7,42,43].

The mechanisms governing commitment to the FR-PTC state remain poorly understood. Current evidence suggests that maladaptive repair reflects a failure to properly complete an initially adaptive repair program rather than the activation of an entirely distinct pathological pathway [7,34,42]. Therefore, persistent epithelial stress provides a cellular substrate for the subsequent development of chronic inflammation and fibrogenic remodeling.

### 4.2. Persistent Inflammation

The resolution of inflammation is a critical component of successful kidney repair. As epithelial integrity is restored, coordinated interactions among epithelial, immune, stromal, and vascular cells normally restore tissue homeostasis in the injured kidney [8,31]. In maladaptive repair, the resolution process remains incomplete.

Persistent inflammation is maintained through continuous bidirectional communication between incompletely repaired epithelial cells and surrounding immune and stromal populations, rather than solely through the prolonged infiltration of immune cells [42,43]. Injury-associated epithelial cells produce chemokines, cytokines, and growth factors that recruit and activate macrophages, lymphocytes, endothelial cells, and fibroblasts. Signals generated by these adjacent cells reinforce epithelial stress and delay the restoration of tubular homeostasis, establishing a self-sustaining inflammatory circuit [7,42,43].

Macrophages mount a highly dynamic response. During the acute phase of AKI, they contribute to the clearance of debris and tissue repair, whereas persistent injury drives heterogeneous macrophage states associated with extracellular matrix remodeling, fibroblast activation, and chronic inflammatory signaling [43]. Recent single-cell analyses indicate that these phenotypes exist along a continuum rather than fitting into the traditional M1/M2 classification, highlighting the importance of the local tissue microenvironment in shaping macrophage function [43].

Adaptive immune cells also contribute to persistent inflammatory signaling through cytokine-mediated interactions with tubular epithelial, myeloid, and stromal cells [43]. Collectively, these multicellular interactions maintain an environment that favors ongoing epithelial dysfunction rather than the restoration of tissue homeostasis.

### 4.3. Fibroblast Activation and Fibrosis

Persistent epithelial injury and unresolved inflammation progressively shift the balance between tissue regeneration and extracellular matrix remodeling. Sustained communication between epithelial, immune, endothelial, and stromal cells promotes fibroblast activation and excessive matrix deposition, leading to interstitial fibrosis [42,43,44,45].

Kidney fibrosis is now recognized as a dynamic multicellular process rather than a simple accumulation of collagen. Injured epithelial cells produce transforming growth factor-β (TGF-β), platelet-derived growth factor, connective tissue growth factor, and other mediators that stimulate fibroblast activation and matrix production [44,45]. Chronic inflammatory signaling and microvascular dysfunction amplify these pathways, thereby creating self-perpetuating profibrotic loops.

Single-cell and spatial transcriptomic studies have substantially reshaped our understanding of kidney fibrogenesis. Activated myofibroblasts arise from heterogeneous stromal populations that acquire distinct transcriptional programs in response to persistent epithelial injury and inflammatory signals [44,45]. Tubular epithelial cells primarily contribute through sustained paracrine communication with surrounding stromal cells rather than through complete epithelial-to-mesenchymal transition, which is now believed to contribute only minimally to the matrix-producing cell population [42,44].

Fibrosis also influences the biological environment in which the surviving nephrons function. Interstitial matrix expansion disrupts the peritubular capillary architecture, impairs oxygen delivery, and limits metabolic recovery, thereby perpetuating epithelial stress and driving maladaptive remodeling [36,44]. Therefore, fibrosis should be regarded as an active component of disease progression rather than a passive accumulation of scar tissue.

### 4.4. Biological Basis of the AKI-to-CKD Transition

The transition from AKI to chronic kidney disease is the cumulative result of persistent epithelial dysfunction, unresolved inflammation, microvascular injury, and progressive fibrogenic remodeling [31,36,42,43,44,45]. Rather than merely reflecting fibrosis, this transition arises from the sustained disruption of interactions among the epithelial, vascular, immune, and stromal compartments.

A defining feature of this transition is the progressive loss of nephron adaptive capacity. Compensatory hyperfiltration by surviving nephrons may initially preserve glomerular filtration but increases metabolic demand, glomerular hypertension, and susceptibility to subsequent injury [46,47]. Concurrently, capillary rarefaction, chronic hypoxia, mitochondrial dysfunction, and persistent profibrotic signaling establish a self-reinforcing cycle that further limits recovery [36,45,47].

The outcome is a kidney that shows apparent functional improvement yet remains biologically different from its pre-injury state. These observations shift the paradigm of recovery from merely restoring filtration to restoring tissue health and tolerance to stress. This biological framework justifies evaluating kidney recovery using markers that reflect persistent cellular injury and tissue remodeling, rather than relying solely on filtration.

### 4.5. The Shift to Biomarker-Based Assessment

Because maladaptive repair may persist despite apparent functional recovery, serum creatinine and eGFR provide only limited information about residual cellular injury and tissue remodeling [31,48]. This limitation has prompted interest in biomarkers that complement filtration-based assessment and may better characterize the biological state of the recovering kidney [48,49]. The key features distinguishing adaptive from maladaptive repair are summarized in Table 2.

## 5. Biomarkers of Biological Kidney Recovery

Most candidate biomarkers of biological kidney recovery were developed or validated in general AKI populations, perioperative or critically ill patients, experimental nephrotoxicity models, or chronic kidney disease rather than specifically in patients recovering from cancer therapy-related AKI [31,42,48,49]. Consequently, their associations with persistent injury or adverse kidney outcomes cannot yet be assumed to translate directly into prediction of rechallenge-associated nephrotoxicity. At present, these biomarkers should therefore be regarded as complementary components of a multidimensional assessment of biological recovery rather than validated determinants of therapeutic rechallenge.

Current biomarkers differ substantially in the biological processes they represent. Some primarily detect ongoing tubular injury or epithelial stress, whereas others complement filtration assessment or provide molecular information about the recovering kidney. Rather than replacing serum creatinine or eGFR, these biomarkers should be interpreted alongside conventional measures to characterize residual injury and the biological state of kidney recovery.

### 5.1. Conventional Assessment of Kidney Recovery

Serum creatinine concentration and eGFR remain cornerstones of clinical assessment after AKI because they are widely available, standardized, and closely associated with clinical outcomes [48,49]. Their main limitation is not a lack of clinical utility, but rather the limited scope of the biological processes they represent. Both primarily estimate glomerular filtration and provide only indirect information about the structural and cellular state of the recovering kidney.

Interpreting these biomarkers is further complicated during recovery from AKI. Serum creatinine is influenced by muscle mass, nutritional status, hydration, and systemic diseases—factors that are particularly relevant in patients with advanced cancer who frequently develop sarcopenia, cachexia, altered fluid balance, and fluctuating nutritional status during systemic therapy [48,49]. Likewise, eGFR equations were developed under steady-state conditions and perform less reliably during the dynamic recovery phase following acute injury [48,50].

### 5.2. Biomarkers of Persistent Tubular Injury

Most currently available biomarkers do not directly measure successful kidney repair. Instead, they identify persistent tubular injury, epithelial stress, or incomplete recovery, which may remain undetected by filtration-based assessments [48,49]. Their value lies in identifying biological processes that continue after the resolution of the acute injury phase.

Kidney injury molecule-1 (KIM-1) is a well-characterized biomarker of proximal tubular injury. KIM-1 is selectively upregulated in dedifferentiated proximal tubular epithelial cells following injury and is minimally expressed in healthy kidneys [51,52]. Persistent elevation of urinary KIM-1 after apparent clinical recovery is generally interpreted as evidence of ongoing epithelial injury rather than of successful repair. Sustained KIM-1 expression has also been experimentally linked to chronic inflammation and fibrogenesis, although its precise role in long-term kidney remodeling remains incompletely understood [51,53].

Neutrophil gelatinase-associated lipocalin (NGAL) reflects acute epithelial stress and is rapidly induced after tubular injury [49,54]. Persistent elevation during the recovery phase has been associated with adverse kidney outcomes in several clinical studies, suggesting ongoing biological activity despite improvements in conventional measures of kidney function. However, interpretation requires caution because systemic inflammation, infection, and malignancy may also increase NGAL concentrations independently of kidney injury [49,54].

Additional biomarkers provide complementary information on specific aspects of tubular injury. Urinary interleukin-18 (IL-18) indicates an inflammatory response, liver-type fatty acid-binding protein (L-FABP) is associated with oxidative and ischemic stress, and the urinary TIMP-2·IGFBP7 reflects cell cycle arrest in stressed tubular epithelial cells [49,55,56]. Each biomarker captures a distinct component of the injury response, but no single biomarker can determine whether epithelial repair is complete. Urinary C-C motif chemokine ligand 14 (CCL14) provides a complementary marker of persistent kidney injury. In the prospective multicenter RUBY study, urinary CCL14 showed good discrimination for persistent severe AKI and outperformed several established functional and injury biomarkers [57]. Its predictive performance was subsequently confirmed in an external validation cohort [58]. These findings suggest that CCL14 may help identify patients at risk of persistent kidney dysfunction rather than directly indicating successful kidney repair. Importantly, however, the supporting evidence is derived primarily from critically ill patients with moderate-to-severe AKI rather than from cancer therapy-related AKI. Therefore, the role of CCL14 in assessing biological kidney recovery or guiding therapeutic rechallenge after cancer therapy-related AKI remains unvalidated.

Therefore, interpretation should focus on the biological processes represented by each biomarker rather than the biomarkers themselves. Persistent abnormalities generally indicate ongoing epithelial stress or injury rather than filtration failure. Conversely, normalization of a single biomarker should not be interpreted as proof that kidney repair is complete. Currently, no injury biomarker reliably distinguishes adaptive repair from established maladaptive remodeling or has been prospectively validated to guide therapeutic rechallenge after cancer therapy-related AKI. Importantly, these biomarkers should not be applied uniformly across different forms of cancer therapy-related kidney injury. KIM-1, NGAL, IL-18, L-FABP, and TIMP-2·IGFBP7 predominantly reflect tubular epithelial injury or stress and may therefore be most informative when tubular damage is a major component of the lesion. In ICI-associated AKI, in which acute interstitial nephritis is the predominant pathological pattern, tubular injury markers may reflect accompanying tubulitis or secondary epithelial injury but do not directly quantify the underlying immune-mediated interstitial process. Similarly, VEGF pathway inhibitor-associated nephrotoxicity primarily involves the glomerular endothelium and podocytes; consequently, tubular biomarkers may incompletely reflect persistent glomerular microvascular injury. In this setting, proteinuria, blood pressure, hematologic evidence of microangiopathy, and, when clinically indicated, kidney pathology remain particularly important for assessing recovery. Thus, biomarker interpretation should be matched to the predominant injury compartment and mechanism rather than applying a uniform biomarker framework across all forms of cancer therapy-related AKI [4,18,21,22,23,24,48,49].

### 5.3. Biomarkers That Complement the Assessment of Functional Recovery

While the biomarkers discussed above primarily reflect persistent tubular injury or epithelial stress, other markers complement the assessment of kidney function and structural integrity. Although none directly measures whether kidney repair is complete, they may improve the interpretation of recovery when integrated with conventional measures of kidney function [48,49].

Among these, cystatin C has attracted considerable interest as an alternative marker of glomerular filtration. Compared with serum creatinine, cystatin C is less affected by skeletal muscle mass and may therefore provide a more reliable estimate of filtration in patients with cancer, who frequently develop sarcopenia or cachexia [59,60]. Cystatin C may also detect acute changes in kidney function earlier than serum creatinine in some clinical settings [61]. However, cystatin C remains a filtration marker. Therefore, it should be viewed as complementary to serum creatinine rather than an indicator of epithelial repair or the restoration of tissue homeostasis.

Proteinuria and albuminuria provide additional information about the recovery of glomerular and tubular integrity. Persistent urinary protein excretion after AKI is associated with adverse long-term kidney outcomes and may indicate ongoing structural abnormalities despite the recovery of filtration [62,63]. However, interpretation requires consideration of the underlying clinical context because proteinuria may reflect persistent glomerular injury, tubular dysfunction, or pre-existing chronic kidney disease rather than merely an active repair process.

### 5.4. Molecular Assessment of Kidney Repair

Emerging molecular approaches, including urinary extracellular vesicles, extracellular RNA profiling, proteomics, and metabolomics, may provide complementary information on epithelial injury, metabolism, inflammatory activity, and intercellular communication [49,64,65].

Single-cell and single-nucleus transcriptomic studies have further identified persistent epithelial populations, including failed-repair proximal tubular cells and related injury-associated states that are not captured by conventional clinical biomarkers [7,34,42]. Translating these molecular signatures into urinary or minimally invasive assays may eventually enable longitudinal assessment of cellular recovery.

However, these approaches remain investigational, with limited standardization and virtually no prospective validation for therapeutic decision-making after cancer therapy-related AKI. Currently available approaches for assessing biological kidney recovery and their clinical applicability are summarized in Table 3.

### 5.5. From Biomarkers to a Clinical Decision Pathway

At present, no validated biomarker threshold can determine whether biological kidney recovery is sufficient for therapeutic rechallenge after cancer therapy-related AKI. In particular, no evidence-based cutoff for urinary KIM-1, NGAL, or other tubular injury markers has been established to mandate continuation, delay, or avoidance of rechallenge. Absolute concentrations are influenced by assay characteristics, sampling time, urine concentration, and the underlying injury phenotype; therefore, a single biomarker value should not be used as a stand-alone decision threshold. Although urinary CCL14 has validated risk strata for persistent severe AKI in critically ill populations (≤1.3, >1.3–13, and >13 ng/mL), these thresholds have not been validated as criteria for therapeutic rechallenge and should not be directly extrapolated to cancer therapy-related AKI [66].

A hypothesis-driven approach may instead integrate serial biomarker trajectories with the clinical and pathological context. After kidney function has stabilized, the predominant injury compartment should first be identified and recovery markers selected accordingly. Persistent or rising injury-marker levels despite improvement in serum creatinine would indicate discordant recovery and should prompt reassessment for ongoing injury. In contrast, declining injury-marker levels accompanied by stable kidney function and resolution of the original injury phenotype would support, but not establish, a favorable biological recovery trajectory.

Conceptually, these findings could define three provisional states: favorable recovery, with concordant clinical improvement and declining or resolved injury signals; uncertain recovery, with discordant or persistently abnormal biological findings; and unfavorable recovery, with persistent or worsening evidence of active kidney injury. These states could inform whether rechallenge is considered, delayed for reassessment, or avoided, respectively, while remaining subordinate to oncologic benefit, kidney reserve, and available treatment alternatives. This framework is hypothesis-generating rather than validated and should be prospectively tested for its ability to predict recurrent nephrotoxicity after rechallenge.

## 6. Therapeutic Rechallenge Following Cancer Therapy-Related AKI

### 6.1. A Practical Framework for Therapeutic Rechallenge

Therapeutic rechallenge following cancer therapy-related AKI is one of the most complex decisions in modern onco-nephrology. Permanent discontinuation of an effective anticancer agent may compromise disease control or eliminate an otherwise valuable treatment option, whereas premature rechallenge may precipitate recurrent kidney injury and accelerate the long-term loss of kidney function [1,2,25]. Therefore, the objective is not simply to determine whether treatment can be resumed, but rather to identify the circumstances under which retreatment is likely to strike an acceptable balance between oncologic benefit and kidney risk.

Rather than relying on a single laboratory threshold, therapeutic rechallenge should be conducted through a structured, stepwise assessment that integrates pathological, biological, oncological, and patient-specific information.

The first step is to confirm the mechanism responsible for the initial kidney injury. Differentiating between direct tubular toxicity, immune-mediated interstitial nephritis, endothelial injury, and unrelated kidney disease establishes the biological context in which retreatment will occur. When the diagnosis remains uncertain and biopsy results are expected to alter clinical management, kidney biopsy should be considered because it can differentiate between lesions with substantially different implications for subsequent treatment [25,28].

The second step is to assess kidney recovery. Recovery should not be judged solely by improvements in serum creatinine or eGFR but should be interpreted in conjunction with the overall clinical course, proteinuria, blood pressure, and electrolyte abnormalities, and, when available, biomarkers reflecting persistent tubular injury or cellular stress [48,49,62,63]. Although no validated biomarker currently indicates successful biological recovery, integrating multiple complementary parameters provides a more informative assessment than relying solely on filtration.

The third step is to estimate oncologic need. The acceptable threshold for kidney-related risk depends on the expected therapeutic benefit. Rechallenge may be justified when the offending agent remains the treatment most likely to improve survival or achieve durable disease control and there are no effective alternatives. Conversely, when comparable treatment options exist or recurrent kidney injury is expected to cause substantial and irreversible kidney impairment, modifying the therapy may be the more appropriate strategy [25,27].

The final step is to plan the rechallenge itself. Decisions go beyond whether or not to restart treatment. They include selecting the same drug or an alternative agent within the same therapeutic class, modifying the dose, optimizing supportive measures, and determining the intensity of post-treatment monitoring. These elements should be tailored to the mechanism of injury, the degree of kidney recovery, and the anticipated therapeutic benefit rather than being applied uniformly to all patients.

**Figure 2:**
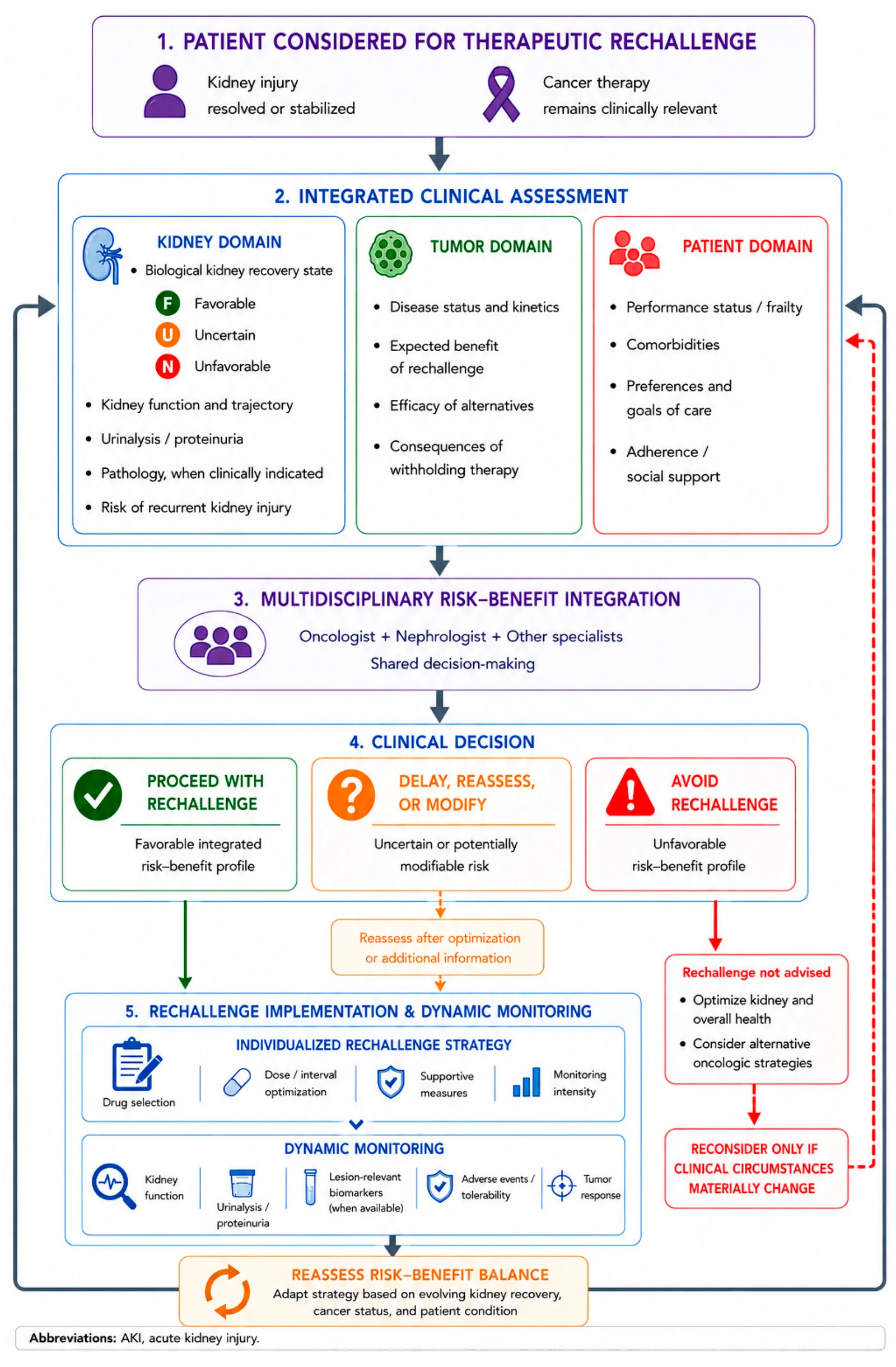
Clinical decision framework for therapeutic rechallenge after cancer therapy-related acute kidney injury. Patients considered for therapeutic rechallenge after cancer therapy-related acute kidney injury (AKI) undergo an integrated clinical assessment across three complementary domains. The kidney domain incorporates the biological kidney recovery state derived from the mechanistic and recovery assessment in Figure 1, together with kidney function and trajectory, urinalysis and proteinuria, pathology when clinically indicated, and the estimated risk of recurrent kidney injury. The tumor domain considers disease status and kinetics, the expected benefit of rechallenge, the efficacy of alternative treatments, and the consequences of withholding therapy. The patient domain incorporates performance status, frailty, comorbidities, treatment preferences and goals of care, and adherence or social support. Multidisciplinary integration of these domains supports shared risk–benefit assessment and leads to one of three provisional clinical strategies: proceed with rechallenge, delay and reassess or modify the strategy, or avoid rechallenge. When rechallenge is undertaken, the treatment strategy is individualized with respect to drug selection, dose and interval, supportive measures, and monitoring intensity. Dynamic monitoring of kidney function, urinary abnormalities, lesion-relevant biomarkers when available, treatment tolerability, and tumor response enables repeated reassessment of the risk–benefit balance and adaptation of the therapeutic strategy as the clinical situation evolves. Figure partially created in BioRender. Taniguchi, H. (2026) https://BioRender.com/c0lxihi (accessed on 25 August 2026). The final illustration was generated and refined using ChatGPT image generation (OpenAI; GPT 5.6-Sol). The scientific concept, content, interpretation, and overall structure were developed and verified by the authors.

This framework should not be interpreted as a formal algorithm or a validated guideline. Rather, it provides a conceptual approach that incorporates our current understanding of kidney injury, repair, and recovery into practical clinical decision-making. Section 6.2.1, Section 6.2.2 and Section 6.2.3 apply these principles to the three major patterns of cancer therapy-related kidney injury for which the available evidence varies substantially: platinum-associated tubular injury, immune checkpoint inhibitor-associated nephritis, and VEGF pathway inhibitor-associated endothelial injury. These principles are integrated into a practical clinical framework for therapeutic rechallenge, as illustrated in Figure 2.

### 6.2. Agent-Specific Considerations for Therapeutic Rechallenge

#### 6.2.1. Platinum-Based Chemotherapy

Therapeutic rechallenge after platinum-associated AKI remains one of the least evidence-based areas in onco-nephrology despite the ongoing importance of platinum compounds in the treatment of many solid malignancies [3,25].

In clinical practice, significant nephrotoxicity frequently results in the permanent discontinuation of cisplatin or a switch to carboplatin. However, there is a lack of prospective data defining appropriate patient selection, timing, or retreatment strategies.

When platinum retreatment is considered, the mechanism of the initial kidney injury must first be determined. Patients with biopsy-confirmed acute tubular injury are fundamentally different from those with persistent interstitial nephritis, glomerular disease, or endothelial injury because the expected mechanisms of recurrent toxicity differ [28]. Kidney biopsy is therefore not routinely required but should be considered when the diagnosis remains uncertain and the result is expected to influence subsequent treatment decisions.

Decision-making should go beyond the recovery of kidney function alone. Baseline kidney reserve, cumulative platinum exposure, prior AKI episodes, concomitant nephrotoxic medications, hydration status, magnesium balance, and the anticipated oncologic benefit all contribute to the overall risk–benefit assessment [3,16]. When retreatment is undertaken, established nephroprotective measures—including adequate intravenous hydration, magnesium supplementation, and the avoidance of additional nephrotoxins—should be routinely incorporated into the treatment plan [3,16,67].

Evidence specifically addressing the renal safety of platinum rechallenge after platinum-associated AKI remains sparse. Retrospective cohorts have demonstrated that platinum retreatment can be oncologically feasible in selected patients with previously treated malignancies [9,10]; however, these studies primarily evaluated antitumor efficacy rather than rechallenge after prior platinum-induced AKI and did not establish kidney-specific eligibility criteria. Renal safety data after a documented episode of platinum-associated AKI remain largely limited to small observational studies and case reports. Our recently reported case of cisplatin administration after recovery from biopsy-proven carboplatin-associated acute tubular necrosis provides one such clinical illustration [11], but should be regarded as hypothesis-generating rather than as evidence of generalizable safety. Accordingly, there remains insufficient evidence to define evidence-based eligibility criteria, optimal timing, or a standardized strategy for platinum rechallenge after AKI. Prospective studies incorporating pathology, longitudinal kidney assessment, and clinical outcomes are needed before standardized recommendations can be developed.

#### 6.2.2. Immune Checkpoint Inhibitors

Among currently available anticancer therapies, therapeutic rechallenge following immune checkpoint inhibitor (ICI)-associated AKI is supported by the most robust clinical evidence [26,27,68].

Although prospective trials are lacking, multiple retrospective cohorts and recent expert recommendations support individualized retreatment after recovery in selected patients when the anticipated oncologic benefit outweighs the risk of recurrent nephritis.

The decision is influenced by several clinicopathologic factors rather than the recovery of kidney function alone. These factors include the severity of the initial AKI episode, the degree of kidney recovery, the presence of biopsy-confirmed acute interstitial nephritis, concomitant immune-related adverse events, and the continued need for immunotherapy [26,27,68]. Potentially nephritogenic concurrent medications, particularly proton pump inhibitors, nonsteroidal anti-inflammatory drugs, and antibiotics, should also be reassessed because the withdrawal of these agents may reduce the likelihood of recurrent immune-mediated injury [18,27].

In the multicenter cohort study reported by Cortazar and colleagues, approximately 22% of patients with ICI-associated AKI were rechallenged, and recurrent AKI developed in 23% of those rechallenged [26]. Importantly, most patients did not develop recurrent nephritis, indicating that ICI rechallenge is not universally contraindicated after AKI [68]. Instead, treatment should be individualized through multidisciplinary discussion involving oncologists and nephrologists while taking into account both kidney safety and the expected oncologic benefit [27,68,69].

Despite the growing body of evidence, important uncertainties remain. No validated biomarker currently predicts recurrent nephritis, and no prospective study has established the optimal timing of retreatment or the role of prophylactic immunosuppression. Consequently, decisions regarding ICI rechallenge continue to rely primarily on toxicity grade, kidney recovery, clinicopathologic assessment, and careful post-rechallenge monitoring rather than on biomarker-guided decision-making [27,48,49,68,70,71]. A biologically informed assessment of kidney recovery may eventually complement these established clinical criteria, but its role in rechallenge decisions requires prospective validation.

#### 6.2.3. VEGF Pathway Inhibitors

Evidence supporting rechallenge after VEGF pathway inhibitor-associated kidney injury remains substantially more limited than that for immune checkpoint inhibitors [21,72]. In many patients, hypertension and low-grade proteinuria can be managed without permanently interrupting treatment. However, nephrotic syndrome, biopsy-proven thrombotic microangiopathy (TMA), and clinically significant AKI represent fundamentally different clinical scenarios in which retreatment requires considerably more caution.

The primary goal is to determine whether endothelial injury has sufficiently resolved to allow re-exposure. Persistent proteinuria, uncontrolled hypertension, laboratory evidence of ongoing microangiopathy, or unresolved kidney dysfunction should prompt a reconsideration of continued therapy. When the diagnosis remains uncertain, kidney biopsy may distinguish kidney-limited TMA from other glomerular lesions or unrelated kidney disease, providing clinically relevant information for subsequent management [28,72].

If retreatment is pursued, close monitoring of blood pressure, urinary protein excretion, kidney function, and hematologic parameters is essential because recurrent endothelial injury may initially present with subtle clinical signs [21,72]. Current recommendations therefore consider VEGF inhibitor rechallenge an individualized, risk-managed strategy reserved for patients with compelling oncologic indications, substantial kidney recovery, and the ability to adhere to close follow-up [72].

Prospective studies are needed to identify clinicopathologic features associated with successful retreatment and to determine whether emerging biomarkers of endothelial injury or kidney recovery can improve patient selection. The available evidence, major factors favoring or arguing against rechallenge, and recommended monitoring strategies for each therapeutic class are summarized in Table 4.

### 6.3. Post-Rechallenge Management

Successful therapeutic rechallenge depends not only on appropriate patient selection but also on careful surveillance after the resumption of treatment. Because recurrent kidney injury may initially present with subtle biochemical or urinary abnormalities, monitoring should be tailored to the initial kidney injury, the nephrotoxic potential of the anticancer agent, and the patient’s residual kidney reserve [25,27,48].

Routine assessment should include serial measurements of serum creatinine, eGFR, and blood pressure, along with urinalysis. Depending on the mechanism of the initial injury, additional assessments may include proteinuria quantification, serum electrolyte monitoring, urine sediment examination, or hematologic assessment for evidence of thrombotic microangiopathy [21,27,72]. Monitoring should be most intensive during the early stages of retreatment, when recurrent toxicity is most likely to become clinically apparent.

Post-rechallenge management should also remain dynamic rather than strictly protocol-driven. Changes in kidney parameters should be interpreted in the context of the oncologic response, cumulative treatment exposure, supportive care measures, and the availability of alternative anticancer therapies. Close collaboration between oncologists and nephrologists facilitates the timely adjustment of treatment intensity, the optimization of renoprotective strategies, and a balanced assessment of competing oncologic and kidney priorities [25,27].

Despite growing recognition of the biologic complexity of kidney recovery, therapeutic rechallenge still relies largely on conventional clinical assessment because no biomarker has been prospectively validated to guide the resumption of treatment after cancer therapy-related AKI [48,49]. The challenge, therefore, is no longer simply determining whether kidney function has recovered, but rather whether kidney repair has progressed sufficiently to tolerate renewed therapeutic stress.

## 7. Future Directions

The major unmet need is to translate mechanistic insights into clinically actionable measures of biological kidney recovery. Future studies should link longitudinal clinical data and kidney pathology with molecular signatures identified through single-cell, spatial, and multi-omics approaches, with particular emphasis on distinguishing adaptive recovery from persistent injury or maladaptive repair [7,34,42,64,65].

Prospective studies should then determine whether such markers improve the prediction of recurrent nephrotoxicity and clinical outcomes after therapeutic rechallenge. This will require standardized biospecimen collection, reproducible analytical platforms, and clinically meaningful endpoints specifically validated in patients with cancer therapy-related AKI. Importantly, biomarker thresholds developed in general AKI populations should not be assumed to define readiness for rechallenge without cancer-specific validation.

**Figure 3:**
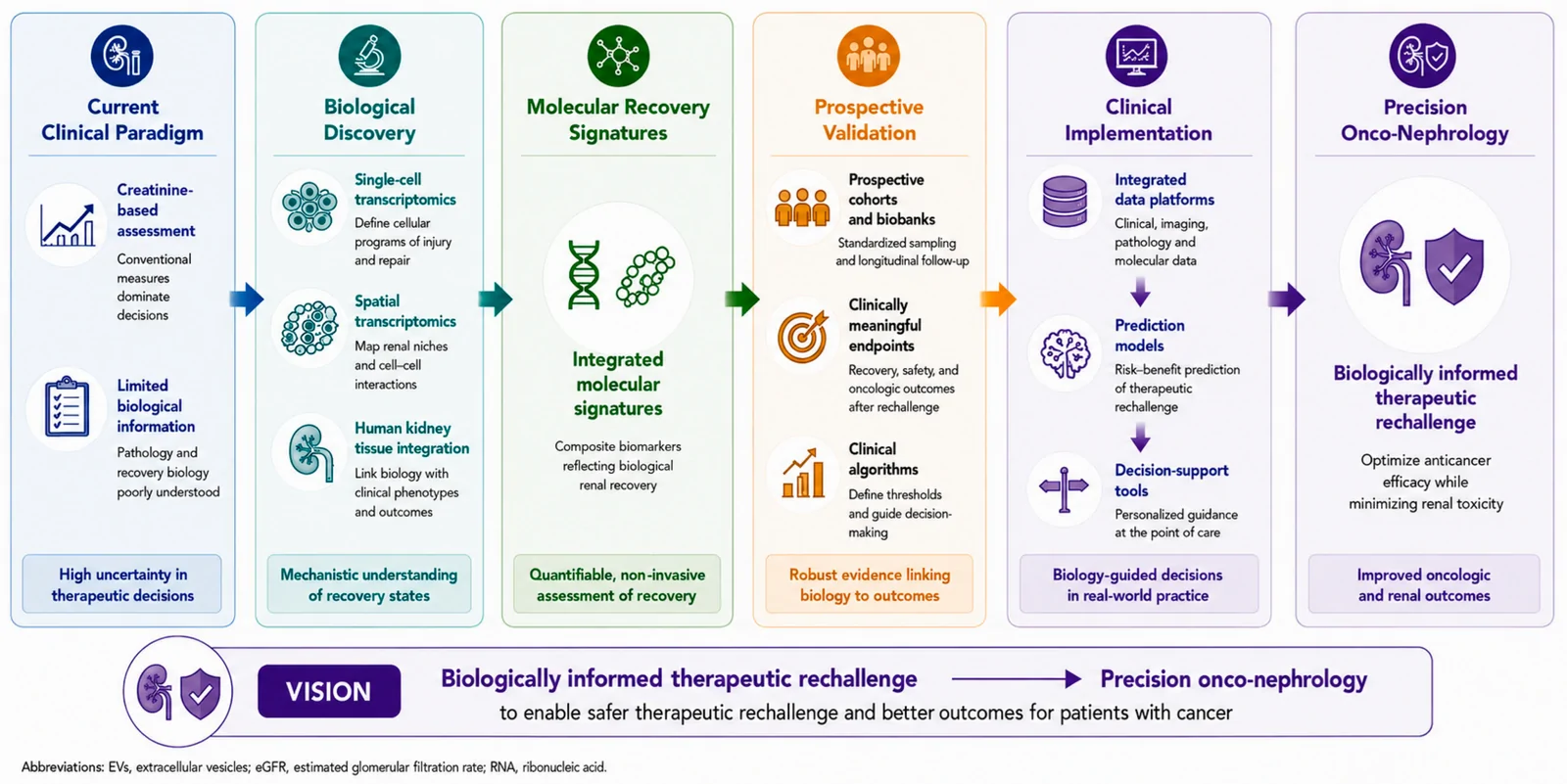
Translational roadmap toward biologically informed therapeutic rechallenge and precision onco-nephrology. Current clinical decision-making after cancer therapy-related acute kidney injury (AKI) remains largely dependent on conventional measures of kidney function and is limited by the lack of validated biomarkers that accurately reflect biological kidney recovery. Future advances are expected to emerge through sequential translational steps, beginning with biological discovery using single-cell and spatial transcriptomics, followed by identification of integrated molecular signatures of kidney recovery through multi-omics approaches. Prospective clinical validation will then establish clinically meaningful recovery endpoints and generate evidence linking biological recovery to patient outcomes after therapeutic rechallenge. Integration of multimodal clinical and molecular data into prediction models and clinical decision-support systems may ultimately enable biologically informed therapeutic rechallenge, supporting the transition toward precision onco-nephrology with the goal of maximizing anticancer efficacy while minimizing renal toxicity. Figure partially created in BioRender. Taniguchi, H. (2026). https://BioRender.com/4obs24i (accessed on 25 August 2026). The final illustration was generated and refined using ChatGPT image generation (OpenAI; GPT 5.6-Sol). The scientific concept, content, interpretation, and overall structure were developed and verified by the authors.

Integration of longitudinal clinical, pathological, and molecular data into multimodal prediction models may ultimately support individualized decision-making rather than replace clinical judgment [73,74]. This translational pathway—from biological discovery to prospective validation and clinical decision support—is summarized in Figure 3.

## 8. Conclusions

Cancer therapy-related AKI has become a growing challenge as advances in systemic anticancer therapy continue to improve patient survival and expand treatment options. Decisions about interrupting treatment or attempting therapeutic rechallenge have traditionally been guided by the normalization of serum creatinine and eGFR. However, these measures primarily reflect filtration and do not necessarily indicate the restoration of epithelial integrity, metabolic competence, microvascular homeostasis, or tissue resilience.

This review introduces biological kidney recovery as a conceptual framework that connects mechanisms of kidney injury, adaptive repair, maladaptive remodeling, biomarker assessment, and therapeutic rechallenge. From this perspective, kidney recovery is not an isolated event but a continuum of biological processes that ultimately determines the kidney’s capacity to tolerate renewed therapeutic stress.

Current clinical practice is still hindered by limited prospective evidence and a lack of validated biomarkers to define successful biological recovery. Nevertheless, integrating injury mechanisms, available pathological findings, conventional measures of kidney function, and emerging biological markers provides a more comprehensive framework for individualized clinical decision-making than relying solely on filtration markers.

Ultimately, the goal is not simply to determine when kidney function has recovered, but to identify when kidney repair has progressed sufficiently to enable the safe and effective continuation of anticancer therapy. Developing biologically informed strategies for therapeutic rechallenge is an important step toward precision onco-nephrology, potentially improving both kidney and oncologic outcomes.

## Figures and Tables

**Table 1:** Major mechanisms of cancer therapy-related kidney injury and implications for therapeutic rechallenge.

Drug Class	Predominant Mechanism	Renal Compartment	Histopathology	Recovery	Rechallenge
Platinum agents	Direct tubular toxicity	Proximal tubule	ATI/ATN	Often reversible	Hydration, dose modification, monitoring
Immune checkpoint inhibitors	Immune-mediated inflammation	Tubulointerstitium	AIN	Immune resolution dependent	Individualized assessment
VEGF pathway inhibitors	Endothelial injury	Glomerular endothelium	TMA	Variable	Monitor proteinuria and blood pressure

Abbreviations: ATI, acute tubular injury. ATN, acute tubular necrosis. AIN, acute interstitial nephritis. TMA, thrombotic microangiopathy. VEGF, vascular endothelial growth factor.

**Table 2:** Cellular trajectories during adaptive and maladaptive kidney repair after acute kidney injury.

Biological Process	Adaptive Repair	Maladaptive Repair	Predominant Evidence Base
Predominant cell state	Transient reparative phenotype	Persistent FR-PTCs	Experimental AKI; supportive human kidney-disease data; direct cancer therapy-related AKI evidence lacking
Cell fate	Cell-cycle re-entry followed by redifferentiation	Persistent cell-cycle arrest and incomplete differentiation	Predominantly experimental AKI
Mitochondrial metabolism	Recovery of oxidative metabolism and mitochondrial homeostasis	Persistent metabolic dysfunction and impaired fatty-acid oxidation	Predominantly experimental AKI and mechanistic kidney-disease studies
Inflammatory response	Resolution of inflammation	Sustained inflammatory signaling	Experimental and general clinical AKI/kidney-disease evidence
Extracellular matrix	Restoration of normal tissue architecture	Progressive extracellular matrix accumulation and fibrosis	Experimental AKI and human kidney-fibrosis studies
Functional consequence	Recovery of tubular integrity and renal resilience	CKD transition and increased susceptibility to recurrent injury	General clinical AKI plus experimental mechanistic evidence; direct evidence for cancer-therapy rechallenge limited
Clinical implication	Greater likelihood of successful therapeutic rechallenge	Higher risk of recurrent nephrotoxicity during rechallenge	Conceptual inference supported by general AKI outcomes; direct cancer therapy–specific rechallenge evidence remains limited

Abbreviations: FR-PTCs, failed-repair proximal tubular cells. CKD, chronic kidney disease. AKI, acute kidney injury.

**Table 3:** Approaches for assessing biological kidney recovery after cancer therapy-related acute kidney injury.

Assessment Domain	Representative Tools	Biological Information Provided	Evidence Base Relevant to Recovery	Current Clinical Applicability
Conventional clinical assessment	Serum creatinine, eGFR, proteinuria, blood pressure	Global kidney function and clinically glomerular or vascular abnormalities	Extensive clinical AKI evidence; routinely used in cancer therapy-related AKI	Standard of care
Kidney pathology	Kidney biopsy	Tubular injury, interstitial inflammation, glomerular/endothelial injury, fibrosis	Direct cancer therapy-related evidence available for selected lesions	Selected patients; defines the predominant injury compartment
Urinary biomarkers	NGAL, KIM-1, IL-18, L-FABP, TIMP-2·IGFBP7, CCL14	Predominantly tubular injury/stress; CCL14 is associated with persistent AKI	Predominantly general clinical AKI and experimental evidence; cancer therapy-specific validation lacking	Emerging; interpretation should reflect the predominant injury compartment
Molecular profiling	uEVs, extracellular RNA, proteomics, metabolomics	Molecular signatures of injury and repair	Predominantly experimental/general kidney-disease evidence	Investigational
Advanced tissue analysis	Single-cell and spatial transcriptomics	Cellular repair states and tissue heterogeneity	Predominantly experimental and human kidney-disease studies	Research

Abbreviations: eGFR, estimated glomerular filtration rate. NGAL, neutrophil gelatinase-associated lipocalin. KIM-1, kidney injury molecule-1. IL-18, interleukin-18. TIMP-2·IGFBP7, tissue inhibitor of metalloproteinases-2 and insulin-like growth factor-binding protein 7. L-FABP, liver-type fatty acid-binding protein. CCL14, C-C motif chemokine ligand 14. uEVs, urinary extracellular vesicles. RNA, ribonucleic acid. AKI, acute kidney injury.

**Table 4:** Practical considerations for therapeutic rechallenge after major patterns of cancer therapy-related kidney injury.

Drug Class	Main Kidney Injury Pattern	Rechallenge Evidence	Factors Favoring Rechallenge	Factors Against Rechallenge	Monitoring After Resumption
Platinum agents	ATI/ATN	Very limited; mainly case-based/observational	Substantial kidney recovery; compelling oncologic benefit; modifiable risk factors controlled	Incomplete recovery; limited kidney reserve; recurrent/severe AKI; ongoing nephrotoxic exposures	Kidney function; electrolytes; hydration status
Immune checkpoint inhibitors	Immune-mediated AIN; less commonly glomerular injury	Most established; retrospective cohorts; recurrent AKI in 23% of rechallenged patients [26]	Substantial recovery; continued oncologic benefit; removal of nephritogenic co-medications	Incomplete recovery/active nephritis; severe initial AKI; significant concurrent irAEs	Kidney function; urinalysis; recurrence of kidney or other irAEs
VEGF pathway inhibitors	Glomerular endothelial/podocyte injury; proteinuria/TMA	Limited; mainly clinical experience/expert guidance	Improved proteinuria/kidney function; controlled BP; compelling oncologic benefit	Nephrotic-range proteinuria; uncontrolled BP; persistent TMA or kidney dysfunction	BP; quantitative proteinuria; kidney function; TMA parameters

Abbreviations: ATI, acute tubular injury. ATN, acute tubular necrosis. AIN, acute interstitial nephritis. TMA, thrombotic microangiopathy. VEGF, vascular endothelial growth factor. AKI, acute kidney injury. irAEs, immune-related adverse events.

## Data Availability

Data sharing is not applicable to this article as no new datasets were generated or analyzed.

## Abbreviations

The following abbreviations are used in this manuscript:

AKI	acute kidney injury
ATP	adenosine triphosphate
CCL14	C-C motif chemokine ligand 14
CKD	chronic kidney disease
CTLA-4	cytotoxic T-lymphocyte-associated protein 4
DNA	deoxyribonucleic acid
eGFR	estimated glomerular filtration rate
FR-PTCs	failed-repair proximal tubular cells
ICI	immune checkpoint inhibitor
IL-18	interleukin-18
KIM-1	kidney injury molecule-1
L-FABP	liver-type fatty acid-binding protein
NGAL	neutrophil gelatinase-associated lipocalin
PD-1	programmed cell death protein 1
PD-L1	programmed death-ligand 1
PGC-1α	peroxisome proliferator-activated receptor-γ coactivator-1α
RNA	ribonucleic acid
SIRT1	sirtuin 1
SOX9	SRY-box transcription factor 9
TIMP-2	tissue inhibitor of metalloproteinases-2
IGFBP7	insulin-like growth factor-binding protein 7
TIMP-2·IGFBP7	tissue inhibitor of metalloproteinases-2 and insulin-like growth factor-binding protein 7
TMA	thrombotic microangiopathy
uEVs	urinary extracellular vesicles
VEGF	vascular endothelial growth factor
Wnt	Wingless/Integrated signaling pathway
YAP	Yes-associated protein
